# Supplementary material for: High-resolution structural and functional retinal imaging in the awake behaving mouse
Source: Commun Biol. 2023 May 29;6:572. doi: 10.1038/s42003-023-04896-x (PMC10227058; doi:10.1038/s42003-023-04896-x)
Supplement: Supplementary file 2 — Supplementary Information [file 42003_2023_4896_MOESM2_ESM.pdf]

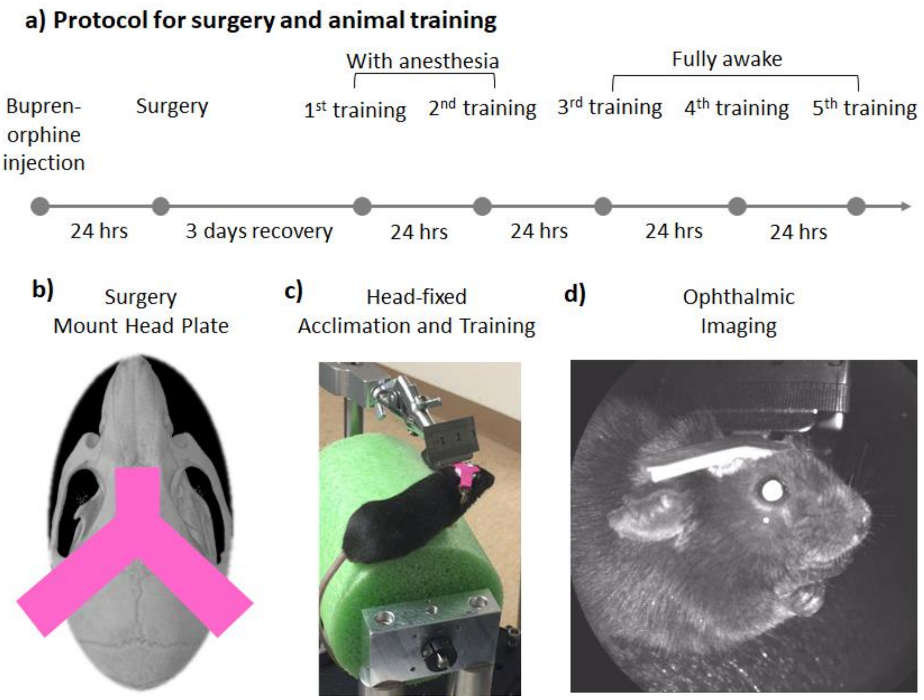

Figure S1. Awake mouse retinal imaging preparation

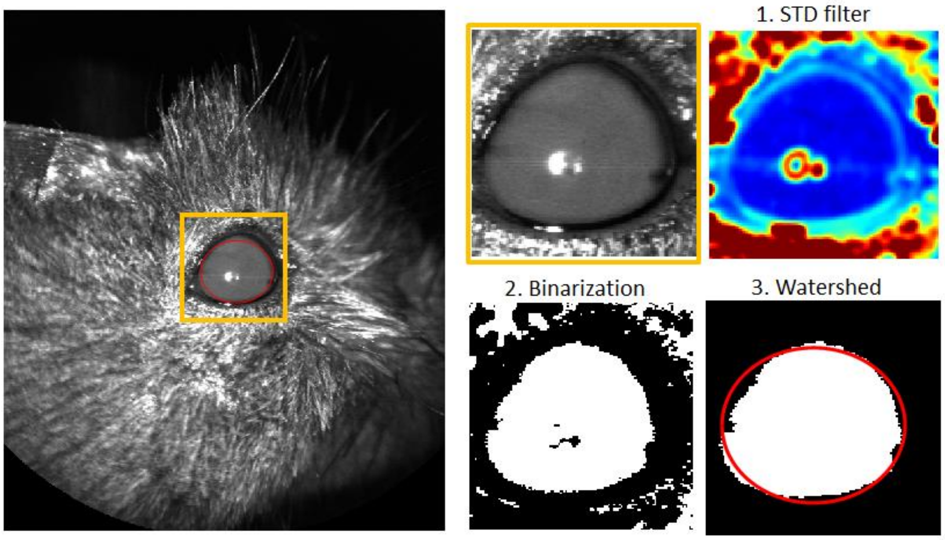

Figure S2. Pupil segmentation

The mouse pupil was imaged with reflectance SLO (790nm, left). To segment and track the pupil, a spatial standard-deviation (STD) filter was applied (step 1). The spatial STD was then binarized to highlight the regions with relatively low spatial variance (step 2). The pupil region

was finally separated from the binary image at step 2 by using watershed algorithms (step 3). The pupil contour was extracted by fitting the binary pupil region with an ellipse function (labelled as red line).

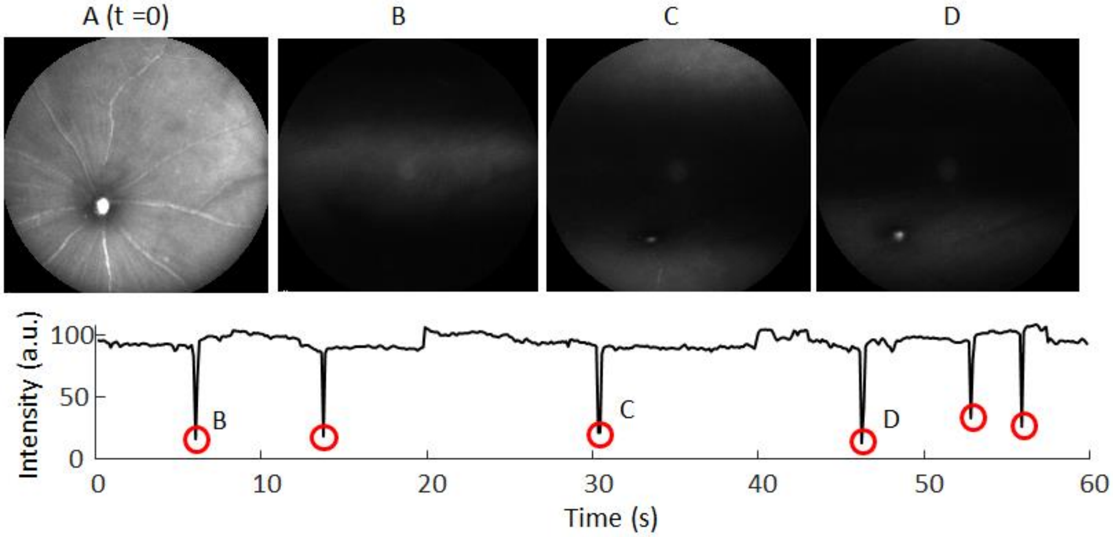

Figure S3. Blink detection

Blinking event was detected from the averaged intensity of each reflectance SLO retinal image (bottom), which blinking can lead to a dramatic reduction of image intensity (labelled as red circles). Typical images with blinks (B-D) are shown in comparison with that with opened eye (A).

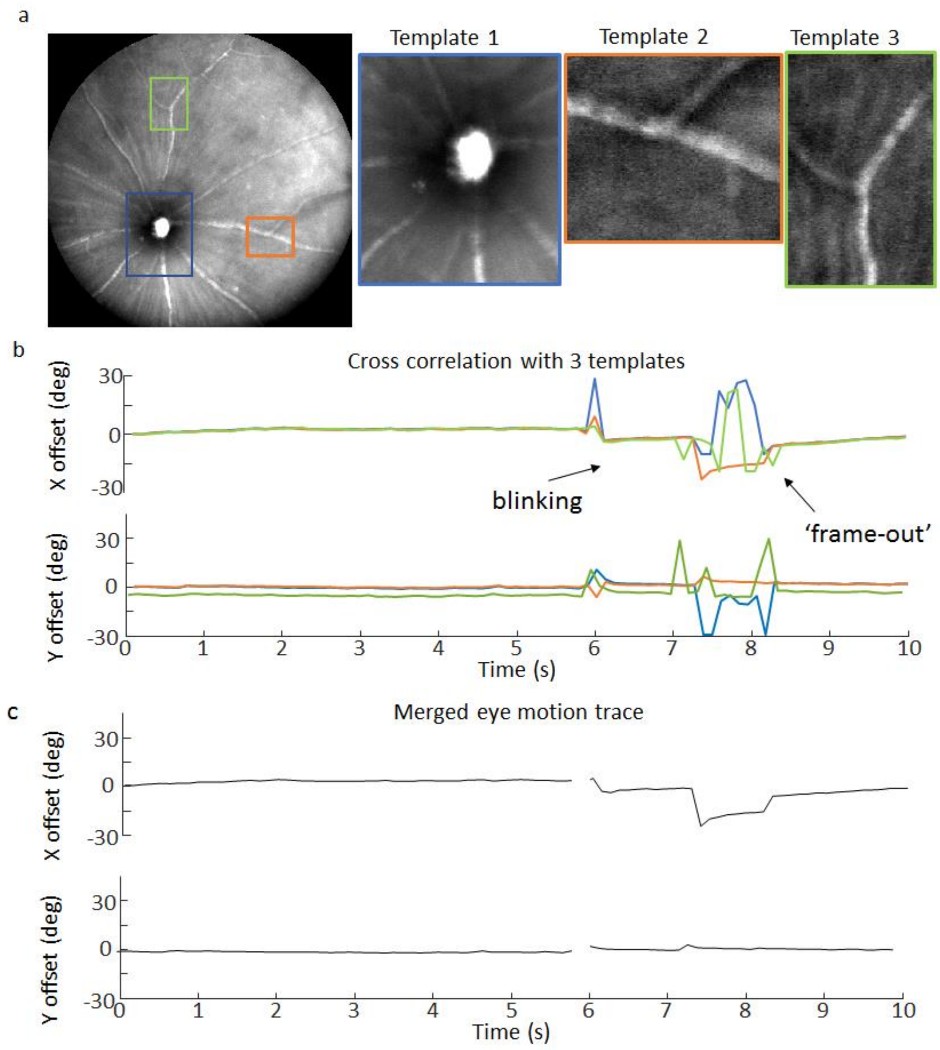

Figure S4. Gaze shift tracking

a) The gaze shift behavior of the awake-behaving mice was measured by tracking the retinal offset in the reflectance SLO imaging (left). Three templates were selected manually as reference for 2D cross-correlation (right). **b)** Extracted candidate traces of retinal offset from 2D cross-correlation with three reference templates. Arrows indicated representative data points with relatively large mismatch which contributed by blinking and 'frame-out' due to large eye motions. **c)** The three motion traces were merged base on the maximum NCC. Data points with blinks were considered 'untrackable' and were removed from the analysis.

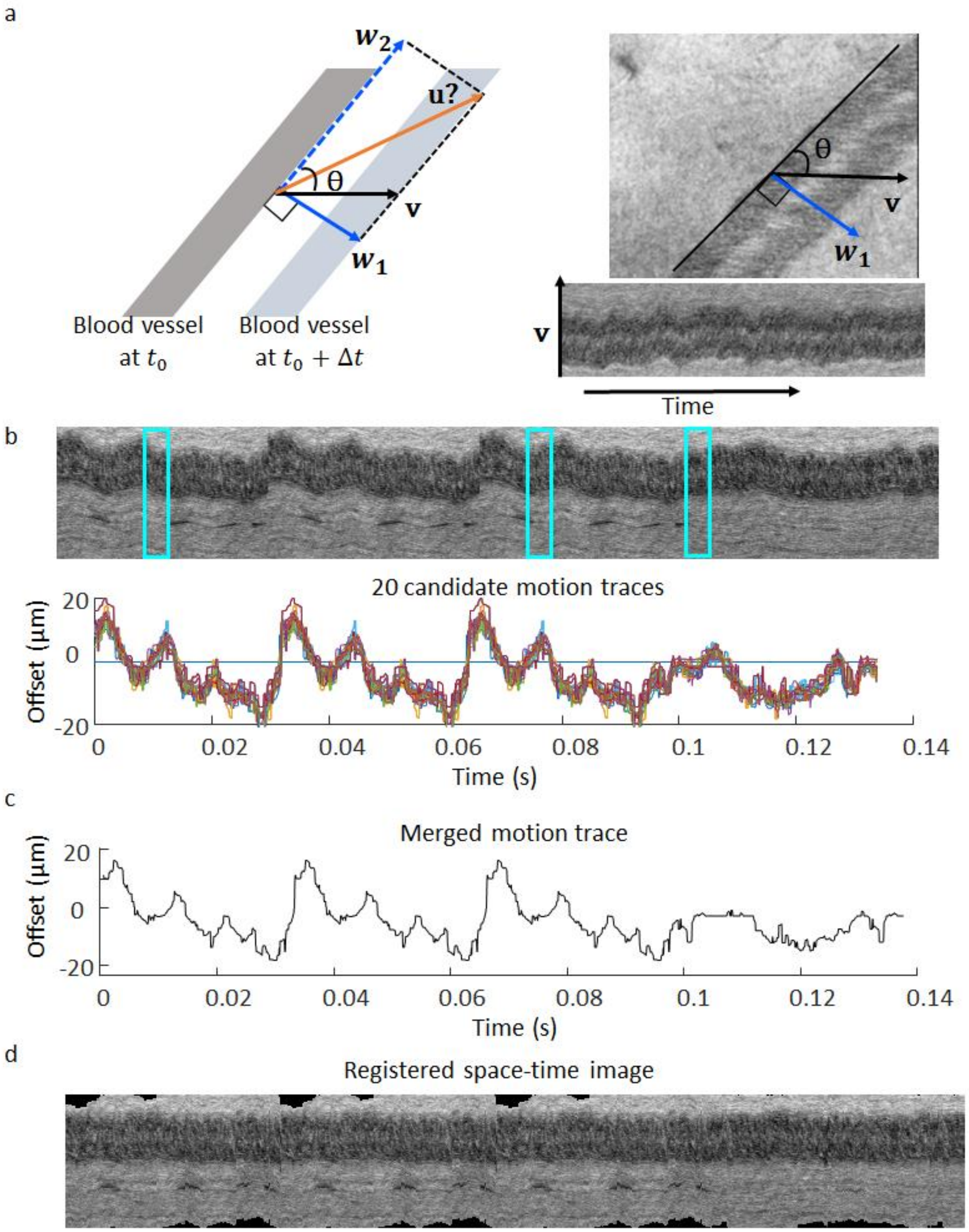

Figure S5. Measurement of high-frequency low-amplitude eye motion

a) When performing a 1D line-scanned AOSLO imaging across a blood vessel, the eye motion at arbitrary direction **u** will induce shearing on the space-time blood vessel profile which is represented as vector **v**. The eye motion vector **u** can be decomposed into two components, $\boldsymbol{w_1}$ which is orthogonal to the blood vessel, and $\boldsymbol{w_2}$ which is parallel to the blood vessel. Information of $\boldsymbol{w_2}$ is unrecoverable due to 1D scanning, while $\boldsymbol{w_1}$ is projected into the shearing **v** the angle $90° - \theta$, where θ is the angle between the scanning axis and the blood vessel. **b)** 20 shearing traces (bottom) extracted by performing 1D cross-correlation strip-by strip on the spatial axis

using 20 randomly selected reference strips (labelled as cyan rectangle in the top panel as examples). **c)** the final motion trace merged from the 20 candidate traces in **b)**. **d)** Registered space-time blood vessel profile for visual examination.

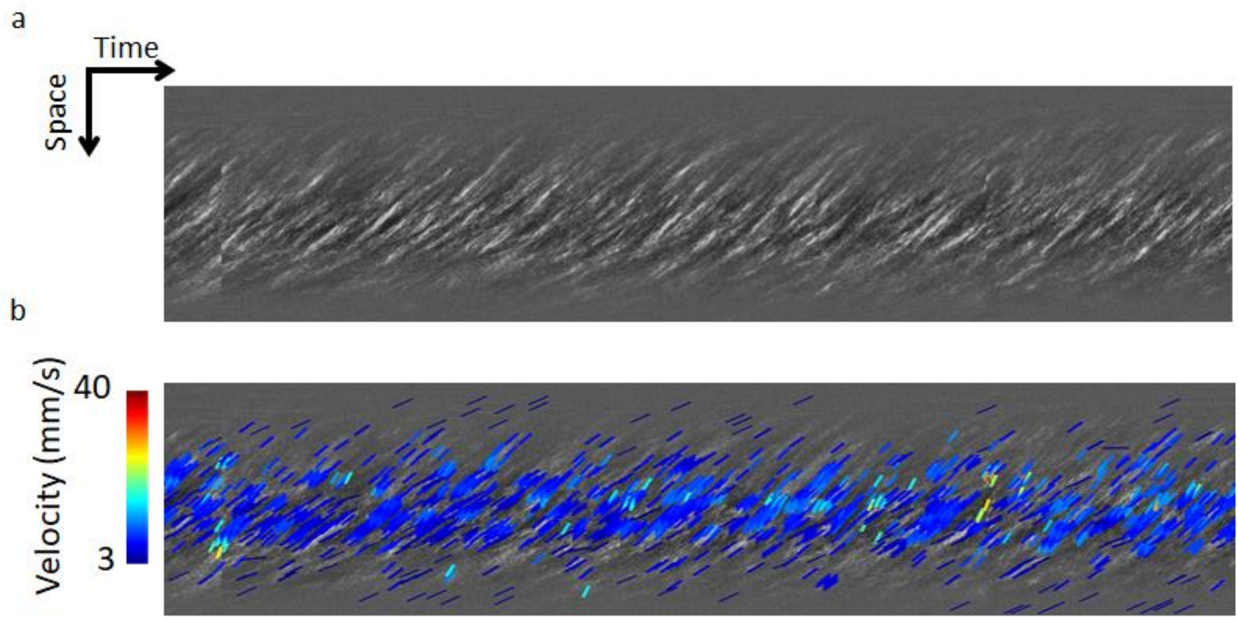

Figure S6. Measurement of blood flow velocity

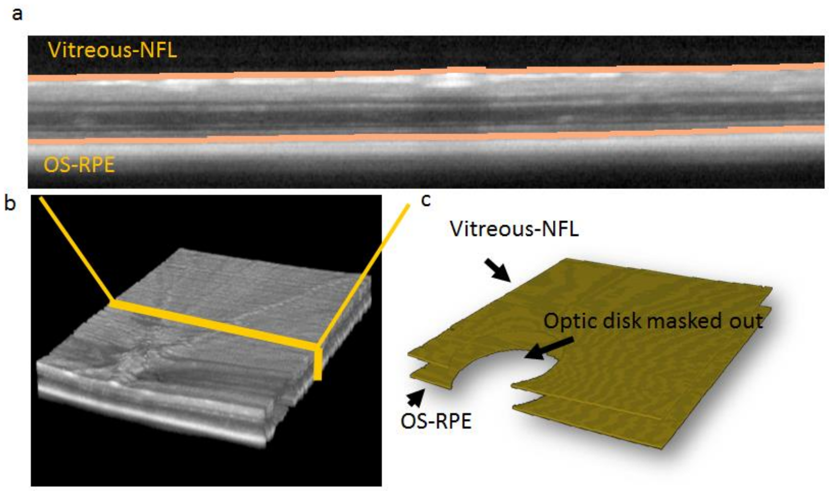

Figure S7. OCT thickness measurement

a) Representative OCT cross-sectional slide with vitreous-NFL boundary and OS-RPE boundary segmented. **b)** An OCT cube imaged from an awake-behaving mouse and **c)** the corresponding 3D segmentation result, the optic disk region was masked out from the segmentation.

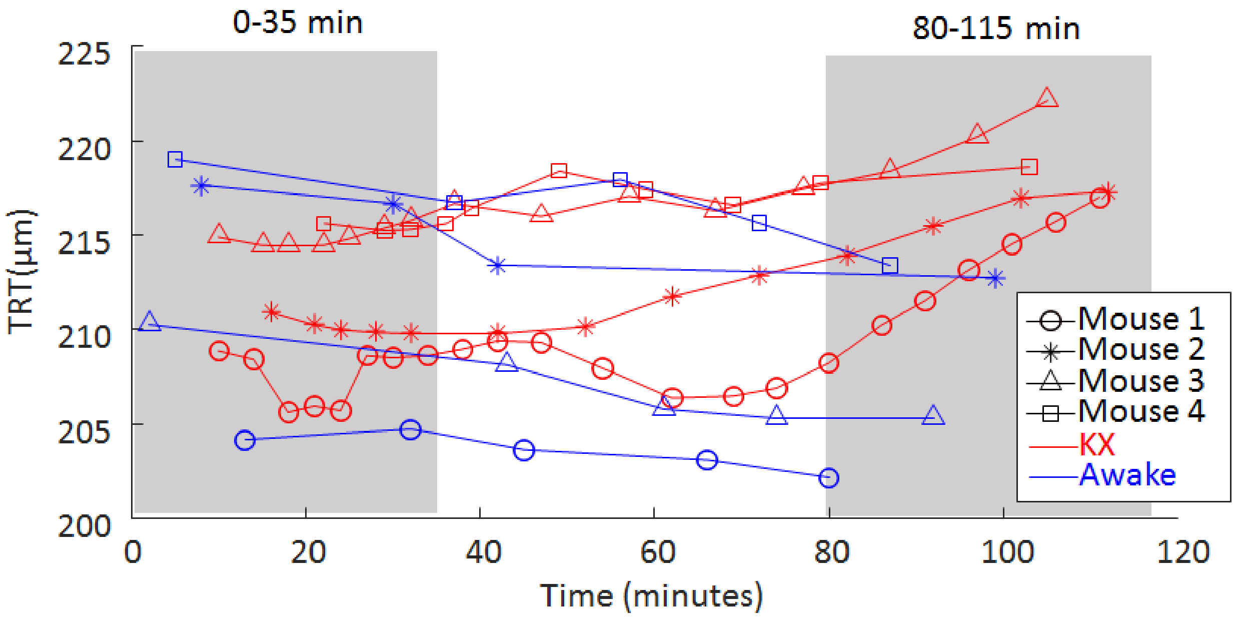

Figure S8. TRT measurement of 4 mice in the awake and anesthetized states

TRT measurement of 4 mice under KX injection and in the awake state for 80-120 minutes. Observations in all 4 mice are identical which the TRT substantially thicken after KX injection while the retinal thickness is relatively stable in the awake state.
