# Supplementary material for: High-resolution structural and functional retinal imaging in the awake behaving mouse
Source: Commun Biol. 2023 May 29;6:572. doi: 10.1038/s42003-023-04896-x (PMC10227058; doi:10.1038/s42003-023-04896-x)
Supplement: Supplementary file 3 — Description of Additional Supplementary Files [file 42003_2023_4896_MOESM3_ESM.pdf]

Description of Additional Supplementary Files

File name: Supplementary Data
Description: The source data behind figures in the paper

File name: Supplementary Video 1
Description: Right: Live video shows the head-restrained mouse preparation. Left: Video shows the grooming behavior of a head-restrained mouse during imaging.

File name: Supplementary Video 2
Description: Video of awake mouse retinal images captured with commercial SLO+OCT platform.

File name: Supplementary Video 3
Description: Live video of 1-minute pupil tracking and pupil persistent analysis.

File name: Supplementary Video 4
Description: Video of 1-minute retina tracking. Top left: live retinal video captured with SLO. Three ROIs used as tracking templates are labeled with rectangles, red indicates the ROI with the highest correlation coefficient. Top right: Live registration montage overlaid with tracked retinal position. Color code indicates time points. Bottom: tracked retinal offsets on horizontal (x) and vertical (y) directions.

File name: Supplementary Video 5
Description: High-resolution AOSLO retinal images of the same blood vessel captured with awake behaving and under anesthesia.

File name: Supplementary Video 6
Description: Motion correction of high-resolution AOSLO images

File name: Supplementary Video 7
Description: 10-hour high-resolution AOSLO imaging of the same ROI.

File name: Supplementary Video 8
Description: Measurement of high-frequency eye motion in the awake mice using line-scanned AOSLO.

File name: Supplementary Video 9
Description: High-frequency eye motion tracked in the same mouse eye over 10 hours.

File name: Supplementary Video 10
Description: High-frequency eye motion tracked in the same mouse eye over 120 minutes after KX injection.
